# Acupuncture Alleviates Anxiety and 22-kHz Ultrasonic Vocalizations in Rats Subjected to Repeated Alcohol Administration by Modulating the Brain-Derived Neurotrophic Factor/Corticotropin-Releasing Hormone Signaling Pathway

**DOI:** 10.3390/ijms22084037

**Published:** 2021-04-14

**Authors:** Su Yeon Seo, Se Kyun Bang, Suk Yun Kang, Seong Jin Cho, Kwang Ho Choi, Yeon Hee Ryu

**Affiliations:** Korea Institute of Oriental Medicine, 1672 Yuseongdae-ro, Yuseong-gu, Daejeon 34054, Korea; ssy1025@kiom.re.kr (S.Y.S.); sichosi@kiom.re.kr (S.K.B.); sy8974@kiom.re.kr (S.Y.K.); ipcng@kiom.re.kr (S.J.C.); ddoongho@kiom.re.kr (K.H.C.)

**Keywords:** acupuncture, USVs, BDNF, CRH, alcohol

## Abstract

The Shenmen point (acupuncture point heart 7: HT7), located in the heart meridian, is frequently used to treat mental disorders, including drug addiction, anxiety, and depression. This study aimed to determine how HT7 regulates anxiety and negative emotions caused by repeated alcohol administration, focusing on the amygdala and paraventricular nucleus (PVN). Repeated administration of alcohol (ETOH; 2 g/kg, i.p. injection, 16% *v*/*v*) for 14 days increased the corticosterone (CORT) levels, and HT7 stimulation reduced the plasma CORT levels. HT7 stimulation mitigated anxiety-like behaviors and reduced 22-kHz ultrasonic vocalizations in rats receiving repeated ETOH injections. HT7 stimulation increased the amygdala expression of mature brain-derived neurotropic factor (mBDNF) and phosphorylated tropomyosin receptor kinase B (pTrkB) and decreased the PVN corticotropin-releasing hormone (CRH) expression. Amygdala microinjections of the TrkB antagonist ANA-12 (0.1 pmol/1 μL) reversed the increase in PVN CRH levels. The reduced PVN CRH levels were regulated by CRH-expressing neurons in the amygdala, and the increased amygdala CRH levels were affected by the HT7-stimulation induced increases in mBDNF. HT7 stimulation alleviates increased stress hormone levels and mitigates anxiety and negative emotions caused by repeated ETOH administration. These results provide scientific support for the clinical use of acupuncture to treat various alcoholism-induced diseases.

## 1. Introduction

According to the statistics from the world health organization, approximately 283 million people have alcohol use disorders. The harmful use of alcohol accounts for 7.1% and 2.2% of the global disease burden for men and women, respectively [1]. Alcohol users exhibit higher rates of comorbid depressive disorders, anxiety, and posttraumatic stress disorder-related neurobehavioral pathologies than do non-alcohol users [2]. In fact, approximately 40% of alcoholic individuals exhibit comorbidities such as anxiety and depressive disorders [3,4].

Various forms of alcohol exposure, including acute and chronic exposure, cause morphological, molecular, and structural changes in the brain [5]. Alcohol administration not only affects mesolimbic system activation but also increases the activity of the hypothalamic–pituitary–adrenal (HPA) axis in rodents [6]. The HPA axis, which is one of the major stress response pathways, has been actively studied in animal models of alcoholism based on the binge/heavy drinking-induced activation of brain stress systems and the chronic relapsing nature of alcohol use disorders [7]. Animal and human studies have demonstrated that an acute alcohol challenge enhances the activity of the HPA axis and pituitary β-endorphin [8]. In contrast to the effects of an acute alcohol challenge, animal studies have demonstrated that chronic alcohol consumption decreases the hypothalamic proopiomelanocortin messenger RNA (mRNA) expression [9]. In addition, activation of the HPA axis following alcohol administration exerts various effects, such as the induction of stress- and addiction-related behaviors [10]. Consequently, increased alcohol consumption induces a negative feedback phenomenon that activates brain stress systems and increases stress hormone levels.

The amygdala is well known as an essential component of the reward system that mediates the stress response and alcohol consumption [11,12]. The amygdala is involved in the attachment of emotional valence to events (i.e., reward and punishment) [13] and is thus crucial for decision making and learning. The expression of brain-derived neurotrophic factor (BDNF) in the amygdala simultaneously represses both anxiety-like behavior and alcohol consumption [14]. A deficiency in BDNF in the neural circuits of the central amygdala (CeA) and medial amygdala (MeA) limits alcohol intake in rats and induces anxiety behaviors derived from BDNF deficiency [15]. The expression of BDNF in the amygdala might be an intervention target in patients with comorbid alcoholism and depression [16], and BDNF thus has therapeutic potential for drug addiction and various mental diseases.

Acupuncture stimulation is an alternative medicine used to treat various drug addictions and emotional disorders, and its effects have been confirmed in clinical studies [17]. Among the acupuncture points, Shenmen (HT7) is the source point of the heart meridian and has frequently been used to treat mental disorders, including drug addiction, anxiety, and depression [18,19]. HT7 stimulation inhibits GABA neuron activity in the ventral tegmental area (VTA) and reduces the self-administration of alcohol [20]. We previously showed that use of the mechanical acupuncture instrument (MAI) at the HT7 point effectively reduces alcohol consumption in rats [21] and that its stimulation regulates immobility and 22-kHz ultrasonic vocalizations (USVs) via σ 1 R in the VTA upon acute alcohol exposure [22]. The stimulation of HT7 reduced alcohol withdrawal-related tremors, anxiety-like behaviors, and alcohol self-administration in alcohol-dependent rats. This effect was determined to be caused by decreased neural activity in the arcuate nucleus and increased β-endorphin levels in the nucleus accumbens [23]. Moreover, acupuncture at HT7 can prevent alcohol-mediated increases in corticotropin-releasing hormone (CRH) mRNA expression in the CeA and the plasma corticosterone (CORT) concentration [24]. This acupuncture point stimulation reduces anxiety-like behaviors by controlling the amygdaloid catecholamine levels during alcohol withdrawal in rats [25]. Acupuncture stimulation at HT7 regulates the HPA axis and increases the BDNF expression in the brain [24,26,27].

Individual studies have investigated the effects of HT7 stimulation on alleviating addiction, anxiety, and depression, but studies on the modulatory potential of the paraventricular nucleus (PVN) and amygdala are insufficient. The main focus of this study was to assess the relationship between CRH and BDNF during sustained alcohol administration and to identify the mechanisms of action of these proteins in the paraventricular nucleus (PVN) and amygdala during HT7 stimulation. Acupuncture at HT7 may represent a potentially novel treatment strategy for alcohol use disorders by activating the BDNF–CRH pathway

## 2. Results

### 2.1. Acute and Repeated ETOH Exposures Increased the Plasma Stress Hormone and ETOH Concentrations

**Figure 1:**
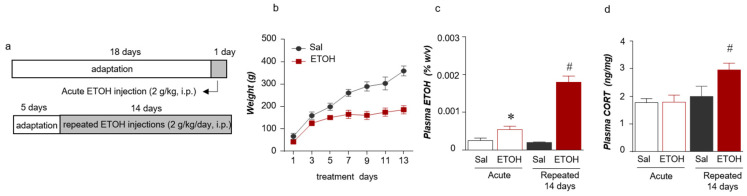
Schematic of the experimental procedure for acute and repeated ETOH administration (**a**). Weight changes were monitored for 13 days (n = 4–5 rats per group) (**b**). Plasma ETOH levels following acute and repeated (14 days) ETOH administration (n = 4–6 rats per group) (**c**). Plasma corticosterone (CORT) levels (n = 6–8 rats per group) (**d**). The data were analyzed using repeated measures one- and two-way ANOVA and t-tests. * *p* < 0.05 compared with the acute Sal group and ^#^ *p* < 0.05 compared with the repeated Sal group. Values are presented as means ± SEM.

We initially measured the blood ETOH levels after the ETOH injections to investigate the effect of ETOH administration (Figure 1a). A smaller increase was observed in the body weight of the ETOH group than in that of the saline (Sal) group. The weight of each group was statistically significantly different on day 7 (treatment (Sal/ETOH): F _(1, 49)_ = 97.12, *p* = 0.0001; days: F _(6, 49)_ = 35.82, *p* = 0.0001; interaction: F _(6, 49)_ = 5.401, *p* = 0.0002) (t = 3.840, df = 7, *p* = 0.0064) and was sustained for up to 13 days (t = 6.150, df = 7, *p* = 0.0005) (Figure 1b). In addition, the acute administration of ETOH increased the plasma ETOH concentration, and the plasma ETOH level increased even after repeated administration for 14 days (treatment (Sal/ETOH): F _(1,16)_ = 79.52, *p* = 0.001; period: F _(1, 16)_ = 32.41, *p* = 0.001; Interaction: F _(1, 16)_ = 38.07, *p* = 0.001) (Figure 1c). The plasma CORT concentration in the repeated ETOH group was significantly increased compared with that of the repeated Sal group. However, the plasma CORT level in the acute ETOH group was not significantly different from that of the Sal group (treatment (Sal/ETOH): F _(1, 20)_ = 4.881, *p* = 0.0390; period: F _(1, 20)_ = 9.944, *p* = 0.0050; Interaction: F _(1, 20)_ = 4.488, *p* = 0.0498) (Figure 1d).

### 2.2. Stimulation at HT7 Reduced the Plasma CORT Levels in the Repeated ETOH-Administered-Rats

**Figure 2:**
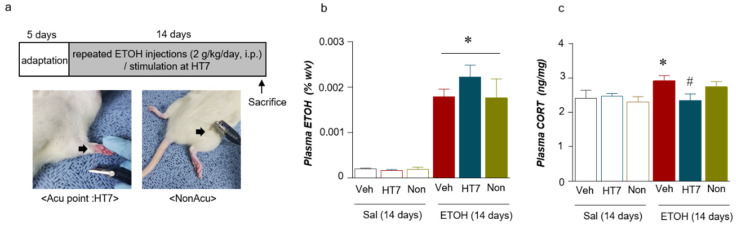
Schematic of the experimental procedure for repeated ETOH administration and representation of the mechanical acupuncture instrument (MAI) treatment (**a**). Plasma ETOH levels after acupuncture stimulation (n = 4–6 rats per group) (**b**). Plasma CORT levels (n = 7–9 rats per group) (**c**). The data were analyzed using repeated measures one- and two-way ANOVA and t-tests. * *p* < 0.05 compared with the Sal + Veh group and ^#^ *p* < 0.05 compared with the ETOH + Veh group. Values are presented as means ± SEM.

Based on the plasma CORT levels, we wished to determine whether stimulation at HT7 decreased the CORT levels and plasma ETOH concentrations in the repeated ETOH-administered rats (Figure 2a). The plasma ETOH concentration in the repeated ETOH- administered group was significantly increased compared with that of the repeated Sal- administered group (Stimulation (Veh/HT7/NonAcu): F _(2, 22)_ = 1.009, *p* = 0.3809; Treatment (Sal/ETOH): F _(1, 22)_ = 146.6, *p* = 0.0001; Interaction: F _(2, 22)_ = 1.244, *p* = 0.3077) (Figure 2b). The plasma CORT concentration in the repeated ETOH-administered group was increased compared with that of the repeated Sal-administered group. The increase in the CORT level induced by repeated ETOH administration was significantly decreased by HT7 stimulation (stimulation (Veh/HT7/NonAcu): F _(2, 51)_ = 3.594, *p* = 0.0347; Treatment (Sal/ETOH): F _(1, 51)_ = 12.79, *p* = 0.0008; Interaction: F _(2, 51)_ = 6.317, *p* = 0.0035) and in the case of ETOH + NonAcu group, there was no statistically significant difference from Veh + ETOH (Figure 2c).

### 2.3. Stimulation at HT7 Alleviated Anxiety-Like Behaviors in Rats Subjected to Repeated ETOH Administration

**Figure 3:**
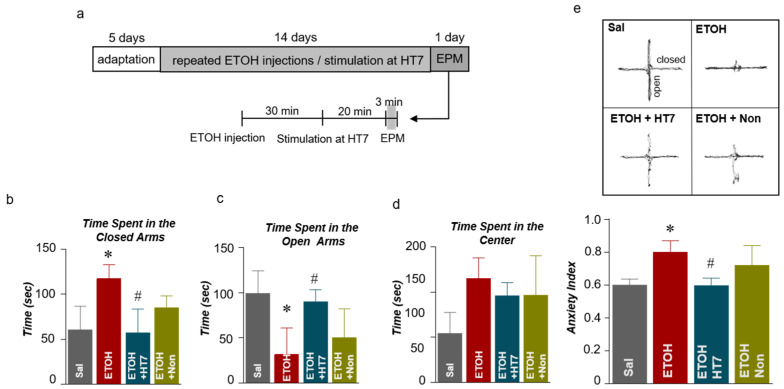
Schematic showing the acupuncture stimulation and elevated plus maze (EPM) test schedule (**a**). Quantification of the number of entries into the closed arms (**b**), open arms (**c**), and center (**d**) of the EPM (n = 10–11 rats per group). Anxiety indexes in the EPM (**e**). The data were analyzed using repeated measures ANOVA followed by Tukey’s test. * *p* < 0.05 compared with the Sal group and # *p* < 0.05 compared with the ETOH group. Values are presented as means ± SEM.

We investigated whether changes in behavior due to ETOH administration were alleviated by stimulation at HT7. The repeated ETOH-administered group was treated with MAI for 14 days, and the anxiety of the rats was recorded 20 min after the MAI stimulation (Figure 3a). In the elevated plus maze (EPM) test, the ETOH group spent significantly more time in the closed arms than did the Sal group, and HT7 stimulation decreased the time spent in the closed arms (F _(3, 31)_ = 5.867, *p* = 0.0027) (Figure 3b). The ETOH group spent less time in the open arms than did the Sal group, but this decrease was alleviated by HT7 stimulation (F _(3, 32)_ = 9.121, *p* = 0.0002) (Figure 3c). Rats in the ETOH group spent more time in the center of the EPM than did rats in the Sal group, but a significant increase was not observed in the HT7-stimulated and NonAcu groups (Figure 3d). The anxiety index was calculated based on the number of visits to the open and closed arms and the center and the time spent in each area. Based on this calculation, the anxiety index increased after ETOH administration and was alleviated by MAI stimulation at HT7 (F _(3, 30)_ = 6.011, *p* = 0.0025) (Figure 3e).

### 2.4. Stimulation at HT7 Reduced ETOH-Induced 22-kHz USVs

**Figure 4:**
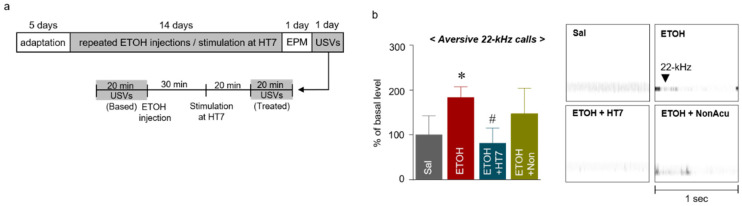
Schematic showing the acupuncture stimulation and schedule for the measurement of ultrasonic vocalizations (USVs) (**a**). Number of 22-kHz USVs emitted during the treatment sessions for 20 min after MAI stimulation (n = 7–9 rats per group) (**b**). The data were analyzed using repeated measures ANOVA followed by Tukey’s test. * *p* < 0.05 compared with the Sal group and # *p* < 0.05 compared with the ETOH group. Values are presented as means ± SEM.

The USVs uttered by rats are widely accepted as an animal model of real-time emotional states [28]. In particular, 22-kHz USVs are considered to be related to inhibition, such as fear, aggression, and freezing [29,30]. Thus, USVs were measured to determine whether MAI stimulation alleviated the emotional changes induced by repeated ETOH administration in rats. Specifically, the USVs were measured for 20 min prior to ETOH administration to determine the baseline value, and the changes in USVs for 20 minutes after ETOH administration and HT7 stimulation were then measured (Figure 4a). The ETOH group showed a significant increase in USVs at 22 kHz compared with that of the Sal group. The USVs at 22 kHz of the rats from the ETOH + HT7 stimulation-treated group were decreased compared with those of rats in the ETOH group (F _(3, 20)_ = 5.001, *p* = 0.0095) (Figure 4b).

### 2.5. Stimulation at HT7 Increased the Levels of mBDNF and Phosphorylated TrkB Receptors in the Amygdala of Rats Subjected to Repeated ETOH Administration

**Figure 5:**
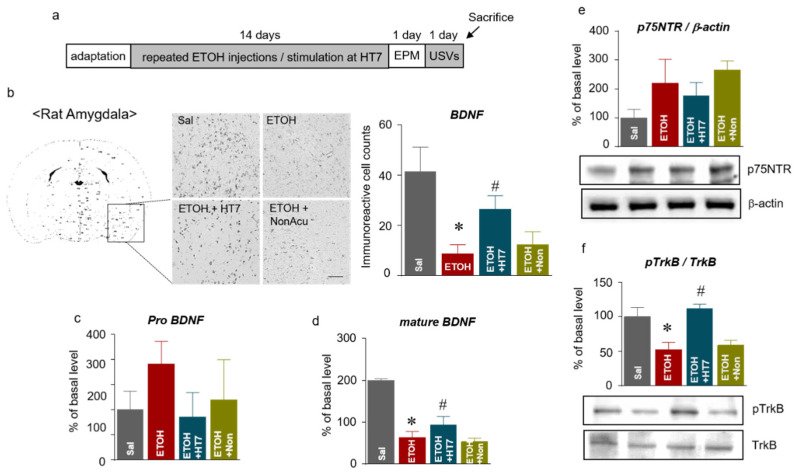
Schematic showing the acupuncture stimulation and sacrifice schedule (**a**). Representative micrographs showing the expression of brain-derived neurotrophic factor (BDNF) in the amygdala. The scale bar represents 100 μm. The results are presented as the number of mature BDNF (mBDNF)-immunoreactive cells (n = 5–7 rats per group) (**b**). The levels of pro-BDNF in the amygdala (n = 46 rats per group) (**c**). The levels of mature BDNF in the amygdala (n = 4–6 rats per group) (**d**). The levels of p75NTR in the amygdala (**e**). The levels of pTrkB in the amygdala (**f**). The data were analyzed using repeated measures ANOVA followed by Tukey’s test. * *p* < 0.05 compared with the Sal group and # *p* < 0.05 compared with the ETOH group. Values are presented as means ± SEM.

We investigated the effect of acupuncture on the BDNF levels in the amygdala of rats subjected to repeated ETOH administrations (Figure 5a). We first performed immunohistochemistry (IHC) to identify the changes in the BDNF levels within the amygdala, and the IHC results showed higher BDNF levels in the amygdala in the ETOH + HT7 group than that in the ETOH group (F _(3, 16)_ = 15.57, *p* < 0.0001) (Figure 5b). Subsequently, ELISA and Western blotting were performed to correctly identify the isoform of BDNF. The levels of pro-BDNF (F _(3, 14)_ = 2.304, *p* = 0.1214) and phosphorylated p75NTR mediated by pro-BDNF (F _(3, 13)_ = 5.411, *p* = 0.0123) were not significantly different among the groups (Figure 5c, e), but the mBDNF level in the ETOH group was significantly reduced compared with that of the Sal group. In contrast, a recovery of the decreased mBDNF levels was observed in the ETOH + HT7 group (F _(3, 15)_ = 108.9, *p* < 0.0001). Additionally, the ETOH + HT7 group showed significantly increased levels of phosphorylated TrkB (pTrkB) compared with that of the ETOH groups (F _(3, 14)_ = 9.360, *p* = 0.0012) (Figure 5d, f).

### 2.6. Stimulation at HT7 Decreased the CRH Levels in the PVN of Repeated ETOH-Administered Rats

**Figure 6:**
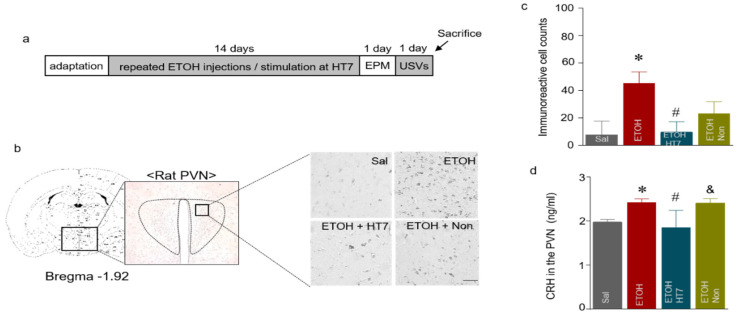
Schematic showing the acupuncture stimulation and sacrifice schedule (**a**). Representative micrographs showing the expression of corticotropin-releasing hormone (CRH) in the paraventricular nucleus (PVN). The scale bar represents 100 μm (**b**). The results are presented as the number of CRH-immunoreactive cells (n = 5–7 rats per group) (**c**). CRH levels in the PVN (n = 4–6 rats per group) (**d**). The data were analyzed using repeated measures ANOVA followed by Tukey’s test. * *p* < 0.05 compared with the Sal group, # *p* < 0.05 compared with the ETOH group, and & *p* < 0.05 compared with the ETOH + HT7 group. Values are presented as means ± SEM.

We observed stress-related behaviors following ETOH administration through EPM and USV measurements, and these behavioral changes are potentially caused by the activation of the HPA axis (Figure 6a, b). Therefore, we analyzed the PVN region using IHC. The levels of CRH in the ETOH group were significantly increased compared with those of the Sal group (F _(3, 25)_ = 14.56, *p* < 0.0001) and ELISA (F _(3, 16)_ = 8.769, *p* = 0.0011) (Figure 6c). The ETOH + HT7 group showed significantly decreased CRH levels compared with those of the ETOH groups (Figure 6d).

### 2.7. The Mechanism by Which Stimulation at HT7 Mitigates the Secretion of CRH in the PVN Is Mediated by BDNF Expression in the Amygdala

**Figure 7:**
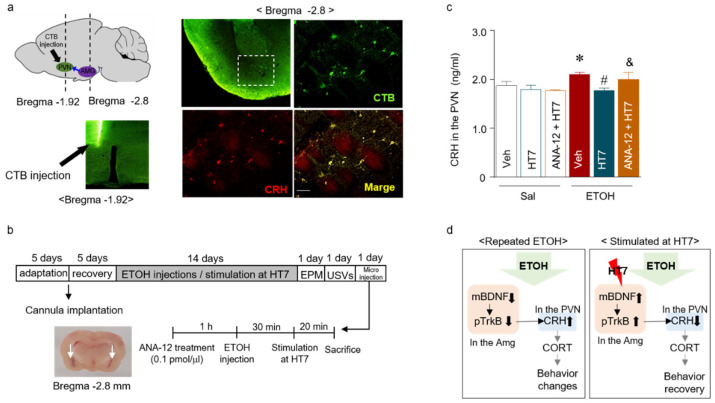
Schematic showing the microinjection protocol and needle placement in the PVN (left panel). CRH-immunoreactive neurons (magenta) and the infusion site of Alexa Fluor 488-conjugated cholera toxin subunit B (CTB-488, green) in the amygdala are shown (right panel). The scale bar represents 100 μm (**a**). Schematic showing the schedule of acupuncture stimulation and microinjection into the amygdala (**b**). CRH levels in the amygdala after pretreatment with the TrkB antagonist (ANA-12, 0.1 pmol/μL) before stimulation of the Shenmen point (acupuncture point heart 7: HT7) (n = 6–8 rats per group) (**c**). The proposed mechanism underlying the relationship between BDNF and CRH and the effect of acupuncture stimulation (**d**). The data were analyzed using repeated measures ANOVA followed by Tukey’s test. * *p* < 0.05 compared with the Sal group, # *p* < 0.05 compared with the ETOH group, and & *p* < 0.05 compared with the ETOH + HT7 group. Values are presented as means ± SEM.

We conducted an experiment to determine whether acupuncture stimulation regulates the secretion of CRH in the PVN by modulating BDNF expression in the amygdala. First, the retrograde tracer CTB-488 was infused into the PVN to assess the CRH-expressing neurons in the amygdala that innervate the PVN. After five days, dense labels were detected throughout the amygdala, which indicated prominent amygdala–PVN afferents to the PVN (Figure 7a, green). In addition, colocalization with CRH-expressing neurons was confirmed by double staining (Figure 7a, magenta). These results showed the presence of afferent neurons from the amygdala to the PVN. Based on this result, the next experiment aimed to determine whether pTrkB decreased the CRH levels following MAI stimulation at HT7. ANA-12 (0.01 pmol) was injected into the amygdala prior to MAI stimulation of HT7 (Figure 7b). CRH expression was significantly decreased in the HT7 + ETOH group compared with that of the Veh + ETOH group, but CRH expression was not significantly decreased in the PVN of the ETOH + ANA-12 + HT7 group (Stimulation (Veh/HT7/NonAcu): F _(2, 22)_ = 8.160, *p* = 0.022; Treatment (Sal/ETOH): F _(1, 22)_ = 11.88, *p* = 0.0023; Interaction: F _(2, 22)_ = 4.128, *p* = 0.0300) (Figure 7c).

## 3. Discussion

In the present study, we elucidated the mechanism by which acupuncture stimulation alleviates the increase in stress hormone levels and mitigates increased 22-kHz vocalizations and anxiety-like behaviors caused by repeated ETOH administration. Based on our findings, MAI stimulation of the HT7 acupoint significantly (1) decreased the plasma CORT level, (2) decreased the anxiety index, (3) decreased aversive 22-kHz USVs, (4) increased the levels of mBDNF and phosphorylated TrkB in the amygdala, and (5) decreased CRH levels in the rat PVN induced by the repeated injection of ETOH for 14 days. Moreover, microinjection of the TrkB receptor antagonist ANA-12 into the amygdala prior to HT7 stimulation significantly suppressed the recovery of the CRH level in the PVN. Thus, anxiety and aversive USVs due to ETOH administration are controlled by the BDNF-CRH system and HT7 stimulation regulates this system by increasing the BDNF level in the amygdala.

Repeated ETOH administration is known to affect the stress system of the body, including the brain [31]. The CORT level increased after the repeated administration of low concentrations of alcohol (2 g/kg, i.p. *v*/*v* 16%) for 14 days. The accumulation of ETOH in the blood increased the levels of stress hormones such as CORT. In general, CORT-treated rats fail to gain weight [32], consistent with the weight changes induced by our repeated ETOH administration protocol. The increased CORT levels might explain why the weight of the rats was reduced by the administration of ETOH.

HT7 stimulation decreases the serum CORT levels in rats with an exogenous CORT-induced memory impairment [33]. As described in studies of various types of alcoholism, including chronic alcohol administration, withdrawal and binge models, HT7 stimulation alleviates depression and anxiety behaviors and alters the plasma CORT levels [16,34]. Consistent with these findings, MAI stimulation at HT7 restored the CORT levels in rats that were repeatedly administered ETOH for 14 days. This acupuncture stimulation reduced anxiety behaviors, as evidenced by the comparisons of various measures, such as the time spent in the open and closed arms, between the MAI-stimulated rats and the repeated ETOH-administered rats. Based on the EPM data, we calculated an anxiety index and found that stimulation at HT7 also mitigated anxiety-like behaviors. Therefore, HT7 stimulation was also effective in a rat model of anxiety induced by repeated ETOH administration.

In general, rodents emit USVs, which represent positive (50–55 kHz) and negative (22–28 kHz) emotions [35]. The 22-kHz USVs are thought to correlate with anxiety in rodents [36]. USV measurements recently revealed an effect of HT7 stimulation, which involves an alleviation of the emotional changes due to administration of various drugs, and the increase in 50-kHz USVs induced by methamphetamine administration is mitigated by HT7 stimulation [37]. We measured USVs to determine whether apparent emotional changes were detected after the induction of anxiety-like behaviors by repeated ETOH administration. The USV results revealed an increase in 22-kHz USVs following repeated ETOH administration, but repeated stimulation at HT7 attenuated these 22-kHz USVs, consistent with the anxiety index calculated from the EPM test data. These findings confirm our hypothesis that HT7 stimulation reduces the increase in anxiety produced by repeated ETOH administration.

Based on the aforementioned EPM and USV data, we identified changes in pathways in the brain following stimulation at HT7. MAI stimulation induced changes in protein levels in the amygdala and PVN. In particular, MAI stimulation at the HT7 acupoint increased the levels of mBDNF and phosphorylated TrkB receptors in the amygdala. Therefore, stimulation at HT7 was closely related to the anti-anxiety effect of BDNF. The CRH is a peptide hormone involved in the stress response. ETOH exposure leads to an increase in CRH signaling. The CRH system is a key regulator of stress-induced alcohol-seeking behavior [38]. According to recent studies, stimulation at several acupuncture points controls the activity of CRH-expressing neurons. For example, electro-acupuncture decreases the hypothalamic CRH levels to the control levels in an animal model of irritable bowel syndrome [39]. The stimulation of various acupuncture points (Qimen, Shenshu, Sanyinjiao, and Zhubin) regulates the glucocorticoid receptor levels and CRH and adrenocorticotropic hormone (ACTH) secretion through the hyperactivation of the HPA axis in the rat model of unpredictable chronic mild stress [40]. As shown in the present study, the increased level of CRH observed after repeated ETOH administration was decreased by HT7 stimulation. Increases in mBDNF levels in the amygdala were accompanied by alterations in the level of CRH in the PVN. However, these results do not establish the signaling relationship between BDNF and CRH. In the following experiments, we identified whether the changes in CRH and BDNF levels induced by MAI stimulation were correlated or independently regulated mechanisms.

Before examining the relationship between BDNF and CRH, we predicted the existence of CRH-expressing neurons connecting the PVN with the amygdala. In fact, CRH-expressing neurons from the central nucleus of the amygdala act on the PVN of the hypothalamus during states of stress [41]. Emotional stimuli activate the HPA axis via the amygdala and forebrain descending pathways. The amygdala excites the hypothalamus to stimulate the HPA axis, functioning as a positive feedback for the excitability of the amygdala by cortisol [42]. Based on this evidence, we used a retrograde tracer to identify CRH-expressing neurons that connect the PVN and the amygdala and thus confirmed that CRH-expressing neurons connect the PVN and amygdala.

Based on the confirmation of the connection between the amygdala and PVN mediated by CRH-expressing neurons, we identified a relationship between BDNF expression in the amygdala and CRH expression in the PVN. When the amygdala TrkB receptor is blocked, HT7 stimulation cannot restore CRH expression in the PVN, indicating that TrkB in the amygdala is phosphorylated during HT7 stimulation and that the CRH expression in the PVN is restored by the phosphorylation of TrkB. Our results suggest a signaling cascade involving BDNF and CRH that is activated in response to MAI stimulation. Stimulation of the HT7 acupoint decreased the release of CRH in the PVN, mediated by phosphorylated TrkB receptors in the amygdala. Therefore, the effect of MAI stimulation at HT7 might be attributed to a change in the pathway that is modulated by the BDNF-CRH system and is related to anxiety-like behaviors and aversive 22-kHz USVs (Figure 7d).

## 4. Materials and Methods

### 4.1. Animals and Treatments

Adult male Wistar rats (250–300 g) were acquired from Orient Bio (Seongnam, Korea). Two rats were housed per cage in an environment with an inverted 12-h light/12-h dark cycle for 5 days, and the humidity and temperature were maintained at 45–55% and 20–23 °C, respectively. On the day of the experiment, the behavioral test was conducted in a quiet room with minimal stress to reduce any confounding variables. During the study period, body weight was recorded daily.

The experiment was conducted to confirm the effect of acupuncture on changes in the behavior and protein levels induced by the intraperitoneal (i.p.) administration of alcohol (ETOH, 2g/kg). ETOH was administered once a day and once a day for two weeks, referring to previous studies (Figure 2a) [43,44].

The acupuncture point HT7 (located on the ulnar aspect of the wrist) and a non-acupuncture point (located on the upper part of the left buttock) were used in this study (Figure 1b). The MAI was used to apply stimulation once daily for 2 weeks. The MAI was developed to provide quantitatively reliable vibration while maintaining the effects of manual acupuncture stimulation. The acceleration parameter of 1.3 m/sec^2^ in intensity and 85 Hz in frequency was routinely used throughout our experiments [45]. This equipment was provided by Daegu Oriental Medical University (Daegu, Korea). The needles (0.18 × 8 mm, Dong Bang Medical, Gyeonggi-do, Korea) continuously stimulated the HT7 point for 30 s, were held in place for up to 1 min after insertion, and then were retracted.

### 4.2. Elevated Plus Maze (EPM) Test

Before the behavioral tests, the rats were placed in a dark room for 30 min, and the EPM test was performed 20 min after the MAI stimulation. This test was performed between 01:00 and 03:00 p.m. in a dimly lit room (±20 lx). The black-coated apparatus was installed 50 cm above the floor. The compartment was divided into two closed arms, two open arms and a center zone, and the rats were able to move freely in all the sections. The distance traveled, time, and the preferred arms were recorded for 3 min after the start of the test (Figure 2a). The test was analyzed using a tracking system (SMART v3.0, Panlab Harvard Apparatus, Barvelona, Spain). The anxiety index value was calculated using the following equation: AI = 1 – ((open arm entries/total arm entries) + (open arm duration/total duration))/2 [46].

### 4.3. Ultrasonic Vocalization

ETOH-administered rats were placed in a 50.8 × 40.6 × 20.3 cm plastic recording chamber within a soundproof room (Gretch-Ken Industries Inc., Lakeview, OR, USA) and USVs were recorded.

Condenser ultrasound microphones (CM16/CMPA, frequency range of 10–200 kHz, Avisoft Bioacoustics, Berlin, Germany) were positioned above the experimental cage without bedding in the recording chamber. USVs were counted through a waveform analysis using Avisoft SASLab Pro. Baseline USVs were measured for 20 min before ETOH administration, and changes in the USVs were measured for 20 min after acupuncture stimulation (Figure 3a). The “50-kHz” USVs were defined as USVs with mean frequencies of 35–70 kHz lasting at least 20–80 ms, and the “22-kHz” USVs were characterized by mean frequencies of 18–32 kHz lasting more than 300 ms [47]. USVs were recorded in real time and stored in a 16-bit format for subsequent analysis (Figure 3b). USVs were analyzed off-line using the Avisoft^®^ SAS LabPro program. The identification of 22-kHz and 50-kHz USVs was performed as described in previous studies [48,49].

### 4.4. Blood ETOH Analysis

For each rat, a 5 mm cut was made in the tail 20 min after the end of ETOH administration. During collection, rats were restrained and kept warm on a heating pad. Tail blood (at least 100 μL) was collected in 200-μL, heparinized capillary tubes and immediately placed on ice. Tubes were centrifuged to separate plasma at the end of the day and stored at −70 °C until analysis. An alcohol assay kit (Cell Biolabs, Inc., San Diego, CA, USA; catalog No. STA-620) was used to identify the levels of the corresponding materials.

### 4.5. ELISA and Alcohol Assay

The pro-BDNF, mature BDNF (mBDNF), CORT, and CRH levels were detected using chemiluminescence with pro-BDNF (Promega Wisconsin, USA; catalog No. BEK- BEK-2217) and mBDNF (Promega; catalog No. BEK-2211) ELISA kits, a CORT ELISA kit (CUSABIO Life Science Inc., Washington, DC, USA; catalog No. CSB-E07014r), and a CRH ELISA kit (CUSABIO; catalog No. CBS-E08038r). The assays were conducted according to the manufacturers’ recommended protocols.

### 4.6. Immunohistochemistry

After the behavioral experiments, the rats were transcardially perfused with phosphate-buffered saline (PBS), and the tissue was fixed using a fixation solution containing 4% paraformaldehyde (PFA) in 0.1 M PBS (pH 7.0). The rat brains were extracted, fixed with neutral buffered formalin 10% (Sigma-Aldrich, Saint Louis, MO, USA), embedded in paraffin, and cut to a thickness of 10 mm to obtain sections. The sections were incubated with the blocking solution for 1 h and then incubated overnight with a 1:500 dilution of a rabbit polyclonal anti-CRH antibody (Invitrogen, Carlsbad, CA, USA; catalog No. PA1-37499). The brain sections were then incubated with a biotinylated secondary goat anti-rabbit antibody followed by an avidin-biotin-peroxidase complex (ABC) (1:500, Vector; catalog No. PK-6101). The ABC complex was visualized, and an image of the section was analyzed using ImageJ software (National Institutes of Health). The average number of CRH-immunoreactive cells per section from each rat was counted and converted into a percentage relative to that of the control group. All quantitative processes and analytical procedures mentioned above were performed blindly by an investigator without knowledge of the experimental conditions.

### 4.7. Western Blotting

Twenty minutes after the final MAI treatment, the rats were anesthetized with N_2_O/O_2_ gas and then sacrificed. The brains extracted from the rats were continuously cut into sections using a rat brain matrix, and the brain regions that were needed for the subsequent analyses were then selected. The brain samples were lysed in radio-immunoprecipitation assay buffer, sonicated for 50 s, and incubated for 1 h on ice. The brain samples were precipitated by centrifugation at 13,000 rpm for 30 min, and the protein contents in the supernatants were assayed using the Bradford method. The samples were electrophoretically separated on 4–10% bis-Tris gels and then transferred to a nitrocellulose membrane. The membrane was blocked with 5% skim milk in Tween-20. Membranes were incubated with primary antibodies overnight at 4 °C. On the next day, the membrane was washed and incubated with secondary antibodies for 1 h at room temperature. All primary antibodies, namely, anti-p75NTR (Cell Signaling Technology, Danvers, MA, USA; Catalog No. 8238S), anti-pTrkB (Abcam; Catalog No. ab109684), anti-TrkB (Abcam; Catalog No. ab18987), and β-actin (Cell Signaling Technology; Catalog No. 4967), were applied at a dilution of 1:1000 in blocking solution in PBS. In addition, the secondary antibody was HRP-labeled goat anti-rabbit or HRP-labeled goat anti-mouse (1:1000, KPL, Gaithersburg, MD, USA). The protein bands on the membrane were exposed to enhanced chemiluminescence reagents (Thermo Fisher). The fluorescence-amplified signals on the membrane were extracted as an image using a Fusion SL4 imaging system (Vilber Lourmat, Eberhardzell, Germany), and the results were quantified using ImageJ software.

### 4.8. Intra-Amygdala Infusion

Each anesthetized rat was fixed in a stereotaxic apparatus, and the skull was exposed. A Hamilton syringe (5-μL Hamilton, Bonaduz AG, Bonaduz, Switzerland) was connected to the infusion pump (UMC4; World Precision Instruments, FL, USA), and 0.1 pmol/1 μL ANA-12 (Tocris, Bristol, UK) or artificial cerebrospinal fluid was injected for 2 min. The needle remained in place for 5 min after the drug injection (amygdala coordinates: posterior, −2.8 mm; lateral, +/−5 mm; deep, −5 mm) [50]. The position of the injection needle was assessed by imaging brain sections, and the infusion concentration and dosage of ANA-12 were determined based on previous studies [51].

### 4.9. Injection of a Retrograde Neuronal Tracer

This experiment was based on the method of retrograde tracing described by Conte [52]. We used a representative retrograde tracer, Alexa Fluor 488-conjugated cholera toxin subunit B (CTB) (Invitrogen, CA, USA), for the experiment, and 0.5 μL of 0.5 mg/mL CTB was injected into the PVN (stereotaxic coordinates: posterior, −1.8 mm; lateral, +/−0.5 mm; deep, −7.8 mm) under anesthesia [53]. Seven days after the injection of this tracer, the anesthetized rats were transcardially perfused with 4% paraformaldehyde (PFA). Fixed brain samples were sliced using a freezing microtome (Leica). The brain sections were visualized using Cell Sens 1.41 software (Olympus Software, Tokyo, Japan).

### 4.10. Statistics

All values are presented as the means ± standard errors of the means. All results (rat body weight, ELISA, ETOH assay, EPM, USVs, Western blot, and immunoreactivity data) were analyzed using one-way or two analysis of variance (ANOVA) with Tukey’s post hoc test. Statistical significance was considered when *p* < 0.05.

## 5. Conclusions

Most treatments for alcoholism aim to reduce alcohol consumption, but few studies have investigated the alleviation of mood disorders caused by alcohol consumption. In the present study, the repeated administration of ETOH to rats increased stress hormone levels, anxiety-like behaviors, and 22-kHz USVs. Stimulation of the HT7 acupoint reversed the increases in stress hormone levels, anxiety-like behaviors, and 22-kHz USVs induced by repeated ETOH administration. These changes were induced by the HT7 stimulation-induced decrease in the CRH release in the PVN, which was mediated by the phosphorylation of TrkB receptors in the amygdala. Thus, acupuncture stimulation alleviates stress and mitigates anxiety caused by ETOH administration. These results provide scientific support for the use of acupuncture in the clinic to treat various diseases caused by alcoholism.

## Data Availability

Not applicable.
